# Re: JACMP Editorial: The meaning of the MS Degree in Medical Physics, Part 4

**DOI:** 10.1120/jacmp.v16i4.5712

**Published:** 2015-07-08

**Authors:** W. A. Beckham, E. F. Jackson

**Affiliations:** ^1^ CAMPEP Commission on Accreditation of Medical Physics Education Programs, Inc. College Park MD USA; ^2^ British Columbia Cancer Agency/University of Victoria Medical Physics Graduate Program Victoria BC Canada; ^3^ British Columbia Cancer Agency Victoria BC Canada; ^4^ Department of Medical Physics University of Wisconsin School of Medicine and Public Health Madison WI USA

To the Editor:

We read with interest the editorial in Volume 16, issue number 2 of the *JACMP* and agree it raises some very important issues in relation to the over‐supply of MS medical physics graduate students attempting to gain entry to clinical medical physics residencies. We concur this is a problem and acknowledge the plight of graduating MS students who find themselves in this position.

In the editorial, candidate #1 suggests graduate programs should be accountable to CAMPEP for their rates of enrolment being similar to the rates at which their graduates are successful at being placed into residency programs. This would imply there are no other viable opportunities for MS graduates other than entry into clinical residency programs. We believe, however, that placement into other forms of nonclinical medical physics employment or starting a PhD degree program are also valid outcomes following MS graduation. Whilst we certainly agree that individual graduate program directors should pay attention to such rates and be transparent with all applicants to MS degree programs by providing them with available data (e.g., annual surveys of graduate and residency programs by CAMPEP) and candid advice, we do not believe that CAMPEP is the appropriate authority to enforce limits on enrolment, except for very specific reasons related to the quality of education and training.

The mission statement from the CAMPEP website reads as follows: “To promote consistent quality education of medical physicists by evaluating and accrediting Graduate, Residency, and Continuing Education programs that meet high standards established by CAMPEP in collaboration with its sponsoring organizations.”

It is clear from this that CAMPEP's mandate is limited to assessment of the quality of education that programs provide, and does not extend to having influence over numbers of students in any given program. The exception would be if a program has too many students or residents for the number of faculty available to adequately support them.

CAMPEP recently introduced its own standards to be met by all accredited programs. For graduate programs, standard 2.11(1) states:^(1)^ “A program must publicly describe the program and the achievements of its graduates and students, preferably through a publicly accessible Web site. This information must be updated no less often than annually and must include, for each degree program (MS and/or PhD), the number of: applicants to the program, students offered admission, students matriculated, and graduates. Where possible, information on the destinations of graduates must also be provided, i.e., residencies, industry positions, etc.”

We encourage students considering entering any MS degree program to look for this information and suggest they only consider applying to programs where it is clear from the publicly disclosed information there is reasonable chance of successful placement into a residency, further degree, or other employment opportunity. If the program does not post the destination information of its graduates on their Web site, then directly ask the Program Director about it.

This advice, unfortunately, may not benefit the current crop of enrolled MS degree students, but hopefully (given this oversupply situation) prospective students will take advantage of it as they research programs to which they apply and ultimately matriculate for pursuit of a terminal MS degree. Students would be well advised to avoid programs that do not exhibit a favorable success rate of its graduates finding desired postgraduation destinations (residency program, higher degree program, or other medical physics employment).

The statements made above are those of the authors. Whilst the authors believe the above information also reflects the opinions of others on the CAMPEP Board based on prior discussions, this letter should not be interpreted as an official CAMPEP position on this matter.
